# Patient Adapted Paternalism for Endomyocardial Biopsy Policy Changes in Heart Transplant Patients: A Mixed-Methods Study

**DOI:** 10.1101/2024.12.29.24319749

**Published:** 2024-12-30

**Authors:** Hyoungmin Kim, Vincenzo Cusi, Melissa McLenon, Rebecca Fielding-Miller, Jose Benjamin Cruz Rodriguez, Jennifer Chak, Marcus Anthony Urey, Paul J. Kim

**Affiliations:** 1 UC San Diego Health, La Jolla, CA.; 2 Herbert Wertheim School of Public Health and Human Longevity Science, University of California San Diego, San Diego, CA.; 3 Center on Gender Equity and Health, School of Medicine, University of California San Diego, San Diego, CA.; 4 Division of Infectious Disease and Global Public Health, School of Medicine, University of California San Diego, San Diego, CA

**Keywords:** Endomyocardial biopsy, acute rejection, heart transplant, patient-provider communication, patient anxiety, interpersonal trust, patient participation, consumer involvement, clinical practice guidelines, shared decision-making, patient adapted paternalism

## Abstract

Endomyocardial biopsies (EMB) are invasive procedures performed in heart transplant (HTx) patients for surveillance of acute rejection. However, patient preferences for replacing EMBs with noninvasive assays in the context of potential institutional policy changes are unknown. A mixed-methods design was used with 28 semi-structured patient interviews and 123 self-administered online survey questionnaires in English and Spanish between January to June 2023. Additionally, we performed semi-structured interviews with 18 HTx team members. Three dominant themes were identified: alleviating patient anxiety and distress, consistent patient-provider communication, and strong interpersonal trust with the HTx team. We found that strong interpersonal trust with the HTx team by the patients was more highly prioritized than their own opinions on whether to replace EMBs with noninvasive assays. Thus, HTx patients often considered surveillance EMBs important to their care (93%), based on the recommendations provided by their HTx team. HTx faculty physicians stated that more multicenter trials are needed prior to replacing EMBs with noninvasive assays. In conclusion, patients identified strong interpersonal trust with HTx team members to justify patient adapted paternalism, where the provider decides in accordance with the patient’s situation, as their preferred shared decision-making paradigm when considering institutional policy on surveillance EMBs.

## 1. INTRODUCTION

‘The end goal is just so much surpassing any kind of inconvenience… Whatever we got to do, I'm on board for it… I mean, I really appreciate how thorough the teams are here.’

The reference standard of detecting acute rejection in heart transplant (HTx) patients is an endomyocardial biopsy (EMB). However, EMBs are invasive procedures with associated complications, such as pericardial effusions, tricuspid valve injury, and inadvertent arterial access.^1^ Thus, noninvasive assays, such as donor-derived cell-free DNA (dd-cfDNA) and circulating microRNAs, are being investigated to improve the HTx patient quality of life.^2–5^

HTx programs vary in their application of noninvasive assays for detection of acute rejection due to a lack of consensus in current HTx guidelines.^2^ This presented a unique opportunity to obtain patients’ perspectives on EMBs at our institution, where policy changes to replace surveillance EMBs with noninvasive assays are being considered.^6^ Seeking perspectives and priorities of patients is critical for development of future clinical practice guidelines, in addition to scientific literature and expert consensus.^3,7,8^ In the current study, we used a mixed-methods approach of adult HTx patients at a single-center to investigate our hypothesis that the majority of patient participants would advocate for changes in our institutional surveillance EMB policy.

## 2. MATERIALS AND METHODS

### 2.1. Data Sharing

The data that support the findings of this study are available upon reasonable request to the authors.

### 2.2. Study Design

This was a prospective, cross-sectional study of HTx recipients who were 18 years of age or older and had undergone at least one EMB. Qualitative and quantitative data were obtained from semi-structured interviews and electronic patient surveys, respectively. The semi-structured interviews were designed to explore patient experiences relating to EMBs and the meanings attributed to these experiences. A simplified set of eight questions (Appendix A) for patients were developed through discussion by the research team (HK, VC, PJK, and RFM) based on prior literature^8,9^ and recent research by the group.^1,5^ We used preliminary results from the qualitative analysis of patient responses to develop a brief self-administered online survey distributed using Qualtrics software (Qualtrics International Inc., Seattle, Washington, USA; Appendix B). The survey was available to patients in English and Spanish–the two most common languages at UCSD. Additionally, the surveys were anonymized to allow for critical and well-defined feedback from patients. Separately, a simplified set of ten questions (Appendix C) for HTx team members were also developed from the qualitative analysis of patient responses. Approval for this study was provided by the UC San Diego Health Office of IRB Administration (No. 805675). This study adheres to the principles of the Declaration of Helsinki formulated by the World Medical Association and the US Federal Policy for the Protection of Human Subjects.

### 2.3. Subject Recruitment

For the semi-structured interviews, a prospective cohort of HTx patients transplanted from January to June 2023 were recruited consecutively. We subsequently invited a separate cohort of HTx patients transplanted from August 2019 to July 2023 to take part in the electronic patient surveys anonymously. All interviewed and invited survey patient participants were eligible to participate in a drawing to win one of ten $50 gift cards. Additionally, we conducted semi-structured interviews of HTx team members, including registered nurses (n = 5), advanced practice providers (n = 4), radiologic technicians (n = 2), and faculty physicians (n = 7), from January to March 2024.

### 2.4. Semi-structured Interview Procedure

Patient interviews were conducted over the phone or in-person using the questions from Appendix A as a guide for each interview. Similarly, HTx team member interviews were conducted in-person using the questions from Appendix C as a guide for each interview. The interviewer (HK, VC, or MM) informed respondents of the purpose of the study and their rights and obtained respondents’ consent to record the interview prior to conducting the interview. Interviews were recorded and transcribed verbatim using Otter.ai transcription software (Otter.ai, Inc., Mountain View, CA, USA) and translated into English if needed. All identifying information was redacted. The research team (HK, VC, PJK, RFM) met weekly until the team determined code and meaning saturation was reached for patient interviews.^10^ For HTx team member interviews, all members were invited to participate.

### 2.5. Data Analysis

We conducted a directed content and thematic analysis with the transcripts independently coded by at least two individuals (HK, VC, or PJK). A finalized codebook was developed and an iterative, thematic process was followed using a modified grounded theory approach.^10–12^ Qualitative analysis of patient interviews was used to guide quantitative analysis of electronic patient surveys.^12^ Patient electronic survey data from all HTx patients were exported and analyzed with descriptive statistics (percentages for discrete responses and median with the interquartile range for continuous responses) using R (R Core Team, 2024).

## 3. RESULTS

### 3.1. Respondent characteristics

Respondent characteristics are shown in Table 1. The number of sensitized patients (panel of reactive antibodies ≥ 10%) was 14.3% and similar to recent literature for US HTx patients.^13^ All EMBs were performed using fluoroscopy guidance, as is institutional practice,^1^ and the majority (86.7%) were performed using the right internal jugular vein as access. The median number of EMB per patient at the time of this study was 8 (IQR, 7 – 10). Most EMBs were performed in an outpatient setting (76.4%) and performed for surveillance indication (83.5%). The median time from HTx to semi-structured interviews was 89 days (IQR, 52 – 172 days).

For the semi-structured patient interviews, 56 participants were invited and 28 (50.0%) respondents agreed to participate. Reasons for non-participation included no response after several attempts (n = 21) and refusal to participate (n = 7) after discussing the purpose of the study. For the patient electronic surveys, 286 participants were invited and 123 surveys were completed (43.0%; Table 2), which include the 28 participants who underwent the semi-structured patient interviews.

For the semi-structured HTx team member interviews, 7 HTx faculty physicians were invited and all agreed to participate. In addition, 5 nurses, 4 advanced practice providers, and 2 radiologic technologists agreed to participate.

### 3.2. Themes

Three major themes were identified: alleviating patient anxiety, consistent patient-provider communication, and strong interpersonal trust between patients and the HTx team (Table 3).

#### Alleviating Patient Anxiety and Distress

‘Music on in the background, and this makes the whole atmosphere much more relaxed.’

HTx patients commonly described anxiety prior to and during invasive EMBs. However, anxiety associated with EMBs decreased with subsequent EMBs in the majority (79.6%) of HTx patients. While a minority of HTx patients described the pain associated with an EMB as ‘inevitable,’ ‘always uncomfortable,’ and ‘something you have to tolerate,’ HTx patients rated their average pain associated with EMBs low at a 3.2 (0 to 10, no pain to the worst pain experienced). Interestingly, the majority of HTx patients (61.5%) that underwent EMBs with either internal jugular vein or brachial vein access preferred the internal jugular vein approach.^14^

HTx patients also provided practical suggestions to alleviate their anxiety and distress associated with invasive EMBs. For instance, HTx patients suggested being allowed to choose their own music that played in the background during the procedure, topical anesthetic before the lidocaine subcutaneous injection, and adjusting sterile drapes to allow patients to see the HTx team members participating in the EMB.

#### Consistent Patient-Provider Communication

‘One thing you guys did [that] was really good was introduce the entire cardiology team on the front end. I [had] nice conversations with everybody. Iťs kind of like a camaraderie.’

HTx patients often cited the importance of consistent patient-provider communication to reduce their anxiety and effectively prepare them for EMBs. Additionally, patients felt the HTx team overall provided honest and open communication. These observations were supported by the fact that the majority (94.8%) of patients indicated that HTx team members did a good job communicating with and comforting patients during the EMB. Most patients (89.4%) also indicated that the EMB results were communicated well to them. In addition, HTx patients indicated that consistent patient-provider communication contributed to the development of interpersonal trust.

HTx patients also provided practical suggestions to further improve patient-provider communication. These suggestions included: more regular updates for unexpected delays for the start of EMBs, reporting of EMB results via the electronic medical record (EMR) portal, similar to other laboratory and diagnostic testing results, and explanation of EMB results in more understandable terms for the patients and their caregivers.

#### Strong Interpersonal Trust with the HTx Team

‘I think that question [adjusting biopsy frequency] should really be up to the cardiologists…The end goal surpasses any kind of inconvenience.’

Patients frequently emphasized strong interpersonal trust with their HTx team. Patients further reported a sense of connection with the HTx team that allowed them to put their trust in the HTx team. Although patients mentioned aspects of EMBs that may be unpleasant, indicated by the 77.9% of HTx patients surveyed preferring a non-invasive alternative to EMBs if available, the theme of strong interpersonal trust with the HTx team was most highly prioritized by the patients. This is also reflected by the fact that 93.0% of survey respondents somewhat or strongly agreed that EMBs are an important part of their post-transplant care.

HTx patients also frequently verbalized their gratitude and feeling indebted to the HTx team when describing their strong interpersonal trust with the HTx team. Comments from the open-ended responses of the patient electronic surveys supported this sub-theme, as the top code identified was the patients’ gratitude to the HTx team (n = 10). Patients who encountered unpleasant EMB experiences typically stated that these issues are minor relative to the exceptional treatment they overall receive from the HTx team. Thus, the majority of patients (91.2%) would endorse changes to the frequency of their EMBs, if the HTx team recommended this change for their post-HTx care.

#### HTx team member perspectives

‘[The patients] become almost like family as we see them so often.’

All HTx team members interviewed (n = 18) were supportive of the improvements to the EMB experience suggested by patients. Providers’ suggestions to improve the EMB experience included: use of smaller facial drapes, giving time for the subcutaneous lidocaine to take effect, encouraging trainees to introduce themselves to the patients, and implementing a waiting room screen that provides on-time or delayed statuses. While most team members agreed that EMB results should be more clearly communicated to patients, patient understanding of EMB results via the EMR portal was a concern brought up by some HTx team members (22%).

Most HTx faculty (83%) supported a reduction in the frequency of surveillance EMBs, though there was no consensus on how much to reduce surveillance EMBs. The majority of the HTx faculty (56%) were hesitant to stop surveillance EMBs completely and stated that more multicenter trials for noninvasive biomarkers were needed.

## 4. DISCUSSION

In the current study, we sought to evaluate our hypothesis that HTx patients would prefer informed patient choice as the shared decision-making paradigm with respect to their EMB-related HTx care. Our modified grounded theory approach revealed three primary themes: 1) alleviating patient anxiety and distress, 2) consistent patient-provider communication, and 3) strong interpersonal trust with the HTx team. Thus, rejecting our initial hypothesis, we found that patients trusted the recommendation of noninvasive assays or invasive EMBs to their HTx team, exemplifying patient adapted paternalism.

Previous studies in other procedural fields have described the importance of alleviating patient anxiety and unnecessary distress. For instance, music therapy is considered to have a positive, moderate effect on reducing patient anxiety among patients who underwent bone marrow biopsies or colonoscopies.^15,16^ Karewicz et al. observed that the reduced anxiety pre-procedure and discomfort during the procedure is related to improved satisfaction post-procedure among patients that underwent bronchoscopies.^17^ HTx patients from our study also provided practical suggestions to reduce their anxiety related to EMBs, including allowing patients to choose their own music during the procedure and utilizing topical lidocaine to reduce the pain associated with subcutaneous lidocaine injections pre-procedure.

Consistent patient-provider communication was also highly emphasized by HTx patients in our study. Wade et al. previously described the importance of communication between patients and providers to better prepare patient’s expectations for transrectal prostate biopsies.^18^ The importance of straight and honest verbal information from the HTx team by patients was also reported by Ivarsson et al.^19^ Additionally, the critical relationship between patient-provider communication and “uncertainty management” has been described by others.^20^ Consistent with prior literature, we report a relationship between patient-provider communication and alleviating patient anxiety and unnecessary distress, such as timely reporting of EMB results to help reduce patient anxiety. However, we found honest and open patient-provider communication not only to be associated with alleviating patient anxiety and unnecessary distress but also to be linked with development of strong interpersonal trust through repeated interactions.^21^

We are the first to report that strong interpersonal trust by patients with their HTx team is closely intertwined with the patients’ perspectives of post-HTx care. As a result, we found patients more highly prioritized their strong interpersonal trust with their HTx team over their freedom to choose a noninvasive option to replace invasive EMBs. Our findings help us to understand why HTx patients directed our team to patient adapted paternalism, where the provider decides in accordance with the patient’s situation, when HTx patients were asked their preference for noninvasive assays versus invasive EMBs.^6^ We also found that interpersonal trust is closely associated with strong affect. Our patients often described their interpersonal trust with the HTx team together with strong feelings of gratitude towards HTx team members. Our findings are consistent with others that have described HTx as the beginning of hope for many patients and thus commonly associated with expressions of gratitude.^21,22^

In the context of strong interpersonal trust with the HTx team, we found that painful EMB experiences were infrequent and did not leave a lasting impact for most HTx patients. In contrast with our study findings, Gutman et al. described cases of lasting patient distress in patients who underwent kidney biopsies.^8^ However, Toal et al. also reported that patients undergoing kidney biopsies felt at ease when the staff performing the biopsy were known and trusted by them already.^23^ The differences in patient experiences for procedures may in part be due to strong interpersonal trust developed pre-procedure. For the patients described by Gutman et al., it is not clear if there were opportunities to develop interpersonal trust with the provider performing the procedure. At our institution, the same group of HTx faculty providers that care for the patients at the time of HTx also perform EMBs, allowing for continuity of care and development of strong interpersonal trust prior to EMBs.^22^

While patients trusted their HTx team on whether noninvasive assays could replace invasive EMBs, HTx faculty providers described the need for more research and could not form a consensus on this subject. Our study findings are consistent with viewpoints of a recently convened expert panel where multicenter clinical trials were recommended to support regulatory endorsement.^3^ Multicenter observational studies (e.g., NCT03695601: SHORE) and randomized controlled trials (e.g., NCT06414603: ACES-EMB) are currently being conducted to address this critical gap in knowledge.

Whether patient adapted paternalism is the preferred shared decision-making paradigm for all HTx care decisions by patients is a question that warrants further study. We hypothesize multiple patient factors, including interpersonal trust, contribute to the shared decision-making paradigm chosen for different HTx decisions. Additional research is also necessary to understand when and how patient-provider interpersonal trust develops within the context of HTx, as prior studies suggest improved patient adherence correlates with strong patient interpersonal trust with their providers.^24^ Thus, we advocate for more qualitative and mixed-methods studies, like ours, that include the views and preferences of patients with respect to HTx care.^25^ We believe these patient-centered studies will contribute greatly to the development of future clinical practice guidelines by considering the perspectives and experience of HTx patients.^26^

Several limitations should be considered when interpreting the results of this study. First, this is a single center study and may not necessarily represent other centers with different patient demographics and experiences. Additionally, our HTx patient population interviewed consisted mostly predominantly of White male patients, similar to other HTx centers in the U.S.^13^ However, our findings provide a critical starting point to inform future studies evaluating HTx patients’ experiences and their trust in the HTx team. Second, response and recall bias are inherent limitations for mixed-methods studies.^27^ We attempted to mitigate the effect of recall bias by conducting semi-structured interviews within one year of HTx. Third, “verbal disjuncts,” or the presence of incongruities between words and expressive gestures was not specifically evaluated for in this study.^28^ During one particular EMB, it was observed by the provider that the patient was shaking and exhibiting external signs of physical distress. Despite this, the patient denied anxiety or pain during the EMB to the provider when asked and also denied anxiety or distress immediately after the EMB, through an independently conducted interview by a research team member. While video studies for qualitative research continue to evolve,^29^ the presence of verbal disjuncts warrants consideration in future studies. Finally, the response rate for the patient electronic surveys was 43.0% and thus may not be fully representative of the HTx patient experience at our institution. However, the study response rate is consistent with other patient electronic surveys.^30^

## CONCLUSION

In conclusion, HTx patients preferred patient adapted paternalism as the shared decision-making paradigm when considering institutional policy on surveillance EMBs. Our study findings may be relevant to other HTx programs considering policy changes for surveillance EMBs as well as future HTx clinical practice guidelines.

## Figures and Tables

**Table 1.** Patient Characteristics for Semi-Structured Interviews (n = 28).

Recipient characteristics	
Age, y, mean (SD)	60.1 (10.4)
Male, N (%)	22 (78.6)
Race	
Asian, N (%)	1 (3.6)
Black, N (%)	2 (7.1)
Native American, N (%)	0
Other Race, N (%)	6 (21.4)
Pacific Islander, N (%)	1 (3.6)
White, N (%)	18 (64.3)
Ethnicity	
Hispanic or Latino, N (%)	3 (10.7)
Recipient BMI, mean (SD)	27.2 (6.6)
Indication for Transplant	
NICM, N (%)	16 (57.1)
ICM, N (%)	11 (39.3)
Re-HTx, N (%)	1 (3.6)
Allosensitization pre-HTx (PRA ≥ 10%), N (%)	4 (14.3)
Durable MCS, N (%)	6 (21.4)
**HTx characteristics**	
Multiorgan transplant, N (%)	4 (14.3)
Induction therapy, N (%)	9 (32.1%)
De novo DSA	5 (17.9%)

BMI, body mass index; CMV, cytomegalovirus; DCD, donation after cardiac death; HTx, heart transplantation; ICM, ischemic cardiomyopathy; MCS, mechanical circulatory support; NICM, nonischemic cardiomyopathy; PHM, predicted heart mass; PRA, panel reactive antibodies

**Table 2.** Patient Electronic Survey Results (n = 123).

Survey question	Survey response Median (Interquartile range)	Percent of responses that somewhat or strongly agree (≥4)
How much pain do you experience during a biopsy? From 0 (no pain) to 10 (worst pain).		
During an average biopsy?	3.0 (1.0–4.0)	-
During your most painful biopsy?	4.0 (2.0–7.0)	-
How much do you agree with the following statements? From 1 (strongly disagree) to 5 (strongly agree).		
Endomyocardial biopsy is an important part of my post-transplant care.	5.0 (5.0–5.0)	93.0%
I initially felt anxious about biopsies, but this improved with experience.	4.5 (4.0–5.0)	79.6%
The physicians/staff do a good job communicating with and comforting me during the biopsy.	5.0 (4.5–5.0)	94.8%
My biopsies are typically on time.	4.0 (3.0–5.0)	71.9%
My biopsy results are communicated well to me.	5.0 (4.0–5.0)	89.4%
Following a biopsy, I feel like I can do my normal activities.	5.0 (4.0–5.0)	86.0%
If there was a non-invasive alternative to biopsy, I would prefer it.	5.0 (4.0–5.0)	77.9%
I would support a change in biopsy frequency based on my team’s recommendations.	5.0 (5.0–5.0)	91.2%
The procedure is more comfortable with certain providers compared to others.	4.0 (3.0–5.0)	70.5%

**Table 3.** Themes With Illustrative Quotes

Theme	Type of Response	Illustrative Quotations
Alleviating Patient Anxiety and Distress (n = 28)	Positive (n = 12)	It’s just an appointment for me. I’m not nervous...rm completely relaxed, almost falling asleep. Because of [the team's] confidence and the way that they address procedures and things that need to be done, is very calming and allows me to have the confidence in the team to proceed with anything they need to get done to make me feel better. Music on in the background, and this makes the whole atmosphere much more relaxed.
	Negative (n = 16)	I get anxious a little bit...I’m always afraid it’s gonna hurt really bad. Take me, knock me out, do it. Don’t tell me [details]. Nervous. Nervous and scared from the shot. Before every biopsy.
Consistent Patient-Provider Communication (n = 28)	Positive (n = 21)	Every time they ask me how are you feeling, how are you doing. They ask you about 10 times during the biopsy. That’s what makes me feel good. One thing you guys did [that] was really good was introduce the entire cardiology team on the front end. I [had] nice conversations with everybody. It’s kind of like a camaraderie. You can [have questions answered] while they’re working on you.
	Negative (n = 7)	It’s not information that I could digest...the information isn’t reviewed. It’s really, if you don’t hear from us, everything’s fine. And that’s kind of weird. I would feel more secure knowing [the biopsy results] through my lab results rather than waiting only for a positive phone call. My apprehension, anxiousness increases when there's a delay. I don’t know exactly when I'll be taken, and that’s just not a good experience.
Strong Interpersonal Trust with the Heart Transplant Team (n = 22)	Positive (n = 20)	I think [determining biopsy frequency] should really be up to the cardiologists...The end goal surpasses any kind of inconvenience. I defer to all my physicians in terms of the recommendations, so I don’t have a comment. I had faith that I was in good hands... I was at peace... I knew that I was being taken care of [and] I was supposed to be right where I was.
	Negative (n = 2)	When they have some difficulties, they have to change out the cables and such, it makes you a little nervous. A fellow came in and they had her finish [the EMB]. I said, you know, just for the future come around and introduce yourself because that would have been nice to know. She was receptive to hearing my comments.

## APPENDIX A: Semi-Structured Interview Questions for Patients

1. In your own words, why are endomyocardial biopsies performed as a part of your post-transplant care?
2. How do you feel about endomyocardial biopsies compared to other routine tests you perform as a part of your post-transplant care, such as echocardiograms? Frequency?
3. What are your thoughts/feelings prior to a scheduled endomyocardial biopsy?
4. What thoughts/feelings do you experience during the endomyocardial biopsy?
5. What are your thoughts/feelings following an endomyocardial biopsy? For instance, how does it affect your activities for the rest of the day and the next day?
6. What are some changes that could be made to improve your experience during an endomyocardial biopsy?
7. Our program is considering further reducing the number of endomyocardial biopsies. What do you think about this potential change? Do you think this may affect the quality of your care? Do you think this will improve your overall post heart transplant experience?
8. How do you hear about your results for your endomyocardial biopsy? Do you have any suggestions for improving how you get results returned to you?

## APPENDIX B: Patient Electronic Survey Questions

How much pain do you experience during a biopsy? From 0 (no pain) to 10 (worst pain).

1. An average biopsy?
2. The most painful biopsy you've had?

Please answer how much you agree with the following statements from 1 (strongly disagree) to 5 (strongly agree).

1. Endomyocardial biopsy is an important part of my post-transplant care
2. I initially felt anxious about biopsies, but this improved with experience
3. The physician/staff do a good job communicating with and comforting me during the biopsy
4. My biopsies are typically on time
5. My biopsy results are communicated well to me
6. Following a biopsy, I feel like I can do my normal activities
7. If there was a non-invasive alternative to biopsy, I would prefer it
8. I would support a change in biopsy frequency based on my team's recommendations
9. The procedure is more comfortable with certain providers compared to others
10. Have you ever had a biopsy where the doctor went through your arm instead of your neck?
11. Which access site did you prefer for your biopsies? (neck vs arm)
12. Is there anything else you'd like to share with us about your experience with endomyocardial biopsy?

## APPENDIX C: Semi-Structured Interview Questions for Heart Transplant Team Members

1. What words would you use to describe endomyocardial biopsies, both from your perspective and what you have heard from patients?
2. Patients in interviews have suggested topical lidocaine, removal of the sterile face drape, and having the ability to choose their own music during an endomyocardial biopsy. What are your thoughts on these suggestions? Do you think these suggestions could be potentially implemented on a regular basis?
3. Patients in interviews have suggested having the endomyocardial biopsy results available on MyChart like the rest of their laboratory results with a short interpretation of the results. If results could be displayed through MyChart with a scripted real-world interpretation, would you think this would be helpful to patients?
4. Patients stated they would feel less anxious and frustrated if they had some way of knowing how much longer they had to wait for their endomyocardial biopsy while in the waiting room. If this could be implemented, would you think this would be helpful to patients?
5. What are you told the purpose of endomyocardial biopsies are for? (question for heart transplant RN coordinators, advanced practice providers, catheterization lab nurses, and radiologic technologists)
6. Are you familiar with the potential risks of endomyocardial biopsies? (for all providers)
7. Do you think patients have an understanding of why endomyocardial biopsies are performed and its potential risks?
8. Do you think patients understand what is expected of them during an endomyocardial biopsy?
9. Do you have any suggestions for improving the endomyocardial biopsy experience for patients?
10. There is current discussion in the heart transplant field about further reducing the frequency of endomyocardial biopsies. What do you think of this?

## Non-standard Abbreviations and Acronyms

dd-cfDNA: donor-derived cell-free DNA
HTx: Heart transplant
EMB: endomyocardial biopsy
EMR: electronic medical record

## References

[1] Cusi V, Vaida F, Wettersten N, Incidence of Acute Rejection Compared With Endomyocardial Biopsy Complications for Heart Transplant Patients in the Contemporary Era. Transplantation. 2024;108(5):1220–1227. 38098137 10.1097/TP.0000000000004882 PMC11042521

[2] Amadio JM, Rodenas-Alesina E, Superina S, Sparing the Prod: Providing an Alternative to Endomyocardial Biopsies With Noninvasive Surveillance After Heart Transplantation During COVID-19. CJC Open. 2022;4(5):479–487. 35187463 10.1016/j.cjco.2022.02.002 PMC8842090

[3] Kobashigawa J, Hall S, Shah P, The Evolving Use of Biomarkers in Heart Transplantation: Consensus of an Expert Panel. Am J Transplant. Published online March 2, 2023. doi:10.1016/j.ajt.2023.02.025 PMC10387364 36870390

[4] Shah P, Agbor-Enoh S, Bagchi P, Circulating microRNAs in cellular and antibody-mediated heart transplant rejection. J Heart Lung Transplant. 2022;41(10):1401–1413. 35872109 10.1016/j.healun.2022.06.019 PMC9529890

[5] Kim PJ, Olymbios M, Siu A, A novel donor-derived cell-free DNA assay for the detection of acute rejection in heart transplantation. J Heart Lung Transplant. 2022;41(7):919–927. 35577713 10.1016/j.healun.2022.04.002 PMC9670834

[6] Sandman L, Granger BB, Ekman I, Munthe C. Adherence, shared decision-making and patient autonomy. Med Health Care Philos. 2012;15(2):115–127. 21678125 10.1007/s11019-011-9336-x

[7] Armstrong MJ, Bloom JA. Patient involvement in guidelines is poor five years after institute of medicine standards: review of guideline methodologies. Res Involv Engagem. 2017;3:19. 29062544 10.1186/s40900-017-0070-2 PMC5623959

[8] Gutman T, Lopez-Vargas P, Manera KE, Identifying and integrating patient and caregiver perspectives in clinical practice guidelines for percutaneous renal biopsy. Nephrology . 2019;24(4):395–404. 29797384 10.1111/nep.13406

[9] Burton D, King A, Bartley J, Petrie KJ, Broadbent E. The surgical anxiety questionnaire (SAQ): development and validation. Psychol Health. 2019;34(2):129–146. 30450974 10.1080/08870446.2018.1502770

[10] Hennink MM, Kaiser BN, Weber MB. What Influences Saturation? Estimating Sample Sizes in Focus Group Research. Qual Health Res. 2019;29(10):1483–1496. 30628545 10.1177/1049732318821692 PMC6635912

[11] Braun V, Clarke V. Using thematic analysis in psychology. Qual Res Psychol. 2006;3(2):77–101.

[12] Vo AV, Majnoonian A, Ni J, Garfein RS, Wishard Guerra A, Fielding-Miller R. Challenges of COVID-19 case investigation and contact tracing in school settings: An initial investigation. J Sch Health. 2023;93(5):353–359. 36938803 10.1111/josh.13308 PMC10484113

[13] Khush K, Hall S, Kao A, Surveillance with dual noninvasive testing for acute cellular rejection after heart transplantation: Outcomes from the Surveillance HeartCare Outcomes Registry. J Heart Lung Transplant. 2024;43(9):1409–1421. 38759766 10.1016/j.healun.2024.05.003

[14] Harwani N, Chukwu E, Alvarez M, Thohan V. Comparison of brachial vein versus internal jugular vein approach for access to the right side of the heart with or without myocardial biopsy. Am J Cardiol. 2015;116(5):740–743. 26150174 10.1016/j.amjcard.2015.05.044

[15] Shabanloei R, Golchin M, Esfahani A, Dolatkhah R, Rasoulian M. Effects of music therapy on pain and anxiety in patients undergoing bone marrow biopsy and aspiration. AORN J. 2010;91(6):746–751. 20510947 10.1016/j.aorn.2010.04.001

[16] Çelebi D, Yılmaz E, Şahin ST, Baydur H. The effect of music therapy during colonoscopy on pain, anxiety and patient comfort: A randomized controlled trial. Complement Ther Clin Pract. 2020;38(101084):101084. 32056820 10.1016/j.ctcp.2019.101084

[17] Karewicz A, Faber K, Karon K, Evaluation of patients’ satisfaction with bronchoscopy procedure. PLoS One. 2022;17(10):e0274377. 36201528 10.1371/journal.pone.0274377 PMC9536568

[18] Wade J, Rosario DJ, Howson J, Role of information in preparing men for transrectal ultrasound guided prostate biopsy: a qualitative study embedded in the ProtecT trial. BMC Health Serv Res. 2015;15(1):80. 25889315 10.1186/s12913-015-0729-z PMC4350900

[19] Ivarsson B, Ekmehag B, Sjöberg T. Heart or lung transplanted patients’ retrospective views on information and support while waiting for transplantation. J Clin Nurs. 2013;22(11–12):1620–1628. 23039262 10.1111/j.1365-2702.2012.04284.x

[20] Kuang K, Wilson SR. A meta-analysis of uncertainty and information management in illness contexts: Uncertainty and information management. J Commun. 2017;67(3):378–401.

[21] Mechanic D, Schlesinger M. The impact of managed care on patients’ trust in medical care and their physicians. JAMA. 1996;275(21):1693–1697. 8637148

[22] Ivarsson B, Ekmehag B, Sjöberg T. Patients’ experiences of information and support during the first six months after heart or lung transplantation. Eur J Cardiovasc Nurs. 2013;12(4):400–406. 23185079 10.1177/1474515112466155

[23] Toal M, Raynor M, McKeaveney C, “Was my kidney biopsy worth it?”-A qualitative phenomenological study of patient experiences and perceived barriers to kidney biopsy. PLoS One. 2024;19(9):e0310358. 39259730 10.1371/journal.pone.0310358 PMC11389898

[24] Saha S, Jacobs EA, Moore RD, Beach MC. Trust in physicians and racial disparities in HIV care. AIDS Patient Care STDS. 2010;24(7):415–420. 20578909 10.1089/apc.2009.0288 PMC3472674

[25] Tong A, Chapman JR, Israni A, Gordon EJ, Craig JC. Qualitative research in organ transplantation: recent contributions to clinical care and policy: Qualitative research in transplantation. Am J Transplant. 2013;13(6):1390–1399. 23648238 10.1111/ajt.12239

[26] Armstrong MJ, Rueda JD, Gronseth GS, Mullins CD. Framework for enhancing clinical practice guidelines through continuous patient engagement. Health Expect. 2017;20(1):3–10. 27115476 10.1111/hex.12467 PMC5217879

[27] Wolderslund M, Kofoed PE, Holst R, Waidtløw K, Ammentorp J. Outpatients’ recall of information when provided with an audio recording: A mixed-methods study. Patient Educ Couns. 2020;103(1):63–70. 31473043 10.1016/j.pec.2019.08.030

[28] Ross H, Abbey S, De Luca E, What they say versus what we see: “hidden” distress and impaired quality of life in heart transplant recipients. J Heart Lung Transplant. 2010;29(10):1142–1149. 20580266 10.1016/j.healun.2010.05.009

[29] Blikstad-Balas M. Key challenges of using video when investigating social practices in education: Contextualization, magnification, and representation. Int j res method educ. 2016;40(5):511–523.

[30] Wu MJ, Zhao K, Fils-Aime F. Response rates of online surveys in published research: A meta-analysis. Comput Hum Behav Rep. 2022;7(100206):100206.

